# Genetic variants of *IGF2BP2* as potential predictors for perineural invasion of prostate cancer in a Taiwanese population

**DOI:** 10.7150/ijms.109770

**Published:** 2025-02-18

**Authors:** Wei-Chun Weng, Yung-Wei Lin, Chung-Howe Lai, Chia-Yen Lin, Yu-Ching Wen, Lun-Ching Chang, Shun-Fa Yang, Ming-Hsien Chien

**Affiliations:** 1 Department of Post-Baccalaureate Medicine, College of Medicine, National Chung Hsing University, Taichung, Taiwan.; 2 Division of Urology, Department of Surgery, Tungs' Taichung Metroharbor Hospital, Taichung, Taiwan.; 3 Department of Nursing, Jenteh Junior College of Medicine, Nursing and Management, Miaoli, Taiwan.; 4 International Master/PhD Program in Medicine, College of Medicine, Taipei Medical University, Taipei, Taiwan.; 5 Department of Urology, School of Medicine, College of Medicine, Taipei Medical University, Taipei, Taiwan.; 6 Department of Urology, Wan Fang Hospital, Taipei Medical University, Taipei, Taiwan.; 7 Graduate Institute of Clinical Medicine, College of Medicine, Taipei Medical University, Taipei, Taiwan.; 8 Division of Urology, Department of Surgery, Taichung Veterans General Hospital, Taichung, Taiwan.; 9 School of Medicine, Chung Shan Medical University, Taichung, Taiwan.; 10 School of Medicine, National Yang Ming Chiao Tung University, Taipei, Taiwan.; 11 Department of Mathematics and Statistics, Florida Atlantic University, Boca Raton, FL, USA.; 12 Institute of Medicine, Chung Shan Medical University, Taichung, Taiwan.; 13 Department of Medical Research, Chung Shan Medical University Hospital, Taichung, Taiwan.; 14 Pulmonary Research Center, Wan Fang Hospital, Taipei Medical University, Taipei, Taiwan.; 15 Traditional Herbal Medicine Research Center, Taipei Medical University Hospital, Taipei, Taiwan.; 16 TMU Research Center of Cancer Translational Medicine, Taipei Medical University, Taipei, Taiwan.

**Keywords:** Insulin-like growth factor 2 mRNA-binding protein 2, Single-nucleotide polymorphism, Perineural invasion, Prostate cancer

## Abstract

Insulin-like growth factor 2 mRNA-binding protein 2 (IGF2BP2), which binds with high affinity to numerous RNA transcripts, is known to promote tumorigenesis and metastasis, including in prostate cancer (PCa). Several case-control studies investigated associations between *IGF2BP2* polymorphisms and cancer progression. However, the effects of *IGF2BP2* genetic variants on clinicopathological progression and biochemical recurrence (BCR) of PCa remain unclear. In this study, we recruited 698 Taiwanese PCa patients who underwent a radical prostatectomy to investigate associations of *IGF2BP2* single-nucleotide polymorphisms (SNPs) with the risk of BCR and clinicopathological progression. Using a TaqMan allelic discrimination assay, we genotyped three *IGF2BP2* SNPs located in the second intron: rs11705701 (G/A), rs4402960 (G/T), and rs1470579 (A/C). Our findings revealed that these* IGF2BP2* SNPs had no significant effect on initial prostate-specific antigen (iPSA) levels or postoperative BCR. However, patients with the rs1470579 A/C genotype exhibited a higher risk of developing perineural invasion (PNI) compared to those with the homozygous A/A genotype. This association was particularly pronounced in patients with an elevated iPSA level (>10 ng/mL). Clinical observations from The Cancer Genome Atlas database showed that elevated IGF2BP2 levels in PCa tissues were significantly associated with higher Gleason scores and exhibited a trend toward correlating with tumor metastasis. In conclusion, our findings highlight that the *IGF2BP2* rs1470579 A>C polymorphism may increase susceptibility for PNI among PCa patients in the Taiwanese population.

## Introduction

Prostate cancer (PCa) is the most commonly diagnosed cancer and the second leading cause of cancer-related deaths among men globally^1^. Clinically, approximately 15% of cases are metastatic at diagnosis, with a 5-year survival rate of 31%^2^. While tumor metastasis traditionally occurs through blood vessels and lymphatic channels, PCa usually exhibits a tendency to invade and grow along prostatic nerves, a phenomenon known as perineural invasion (PNI). This invasion extends from the prostate to the pelvic plexus^3^. The perineural space was identified as a specialized microenvironment that facilitates both the spread and growth of PCa^3^, ^4^. Furthermore, studies have linked PNI to higher surgical Gleason scores and an increased risk of biochemical recurrence (BCR)^5^-^7^. For instance, a meta-analysis involving 13,412 PCa patients revealed that those with PNI had a 1.4-fold higher risk of BCR following a radical prostatectomy (RP)^8^. Historically, clinical diagnoses relied on digital rectal examinations and measuring blood levels of prostate-specific antigen (PSA) for PCa screening. However, these methods cannot reliably differentiate aggressive tumors based solely on biopsy results and PSA levels^9^. To date, biomarkers for predicting metastatic PCa (mPCa) remain under investigation, with limited studies and insufficient evidence supporting their clinical utility.

Numerous cancer research studies have confirmed the utility of tumor-associated genetic aberration-based biomarkers in assessing risk, enabling early diagnosis, and predicting therapeutic outcomes. Genetic polymorphisms, which refer to variations in genomic sequences among individuals, occur in approximately 1% of the general population. Among these, single-nucleotide polymorphisms (SNPs) are the most frequently observed variations in repeated sequences. Recently, a growing body of research has emphasized the critical roles of SNPs and other genomic alterations in predicting, prognosticating, and determining pharmacotherapeutic outcomes in PCa^10^, ^11^. For example, a key regulator of androgen receptor variants, Y-box-binding protein-1, linked to resistance to androgen deprivation therapy (ADT) in PCa, possesses an intronic SNP (rs1203072) that influences gene expression and is associated with PCa metastasis^12^. Additionally, 14 SNPs across six genes—*XRCC4*, *PMS1*, *GATA3*, *IL13*, *CASP8*, and *IGF1*—were found to be significantly correlated with cancer-specific survival in patients with mPCa^13^. Genetic variants in *ADAM9* were also suggested to be potential predictors of BCR in PCa patients undergoing an RP^14^. Furthermore, SNPs such as rs12422149, rs1789693, and rs1077858 in androgen transporter genes, including *solute carrier organic anion transporter family member 2B1*, were identified as potential pharmacogenomic markers for resistance to ADT in PCa^15^.

The insulin-like growth factor 2 (IGF2) mRNA-binding protein 2 (IGF2BP2), encoded by the *IGF2BP2* gene, functions as an RNA-binding protein for *IGF2* messenger (m)RNA^16^. IGFBP2 expression is generally sustained postnatally and plays a crucial role in RNA localization, stability, and translation. Recent research identified IGF2BP2 as a reader of N6-methyladenosine (m6A), the most prevalent internal RNA modification in eukaryotic cells. IGF2BP2 interacts with various types of RNAs, including mRNAs, circular (circ)RNAs, microRNAs (miRNAs), and long non-coding (lnc)RNAs, regulating diverse disease processes such as diabetes and cancers^17^-^19^. Genome-wide association studies (GWASs) have identified a cluster of SNPs within the second intron of *IGF2BP2* as being associated with type 2 diabetes (T2D). Among these, rs4402960 and rs1470579 are the most frequently reported SNPs across various ethnic groups, including Chinese Han^20^, Lebanese^20^, ^21^, and Indian^22^ populations. In cancer research, the *IGF2BP2* SNPs rs4402960 and rs1470579 were linked to an increased risk of developing breast cancer^23^ and esophageal cancer^24^. Additionally, the SNPs rs11705701, rs4402960, and rs1470579 were associated with advanced clinical stages, larger tumors, and lymph node metastasis in oral cancer^25^. Furthermore, rs4402960 and rs6769511 were identified as strong predictors of the chemotherapeutic response in patients with metastatic gastric cancer^26^. While several studies explored the clinical significance of *IGF2BP2* SNPs in various cancer types, their impacts on PCa remain unexplored. In this study, we investigated associations of SNPs in the *IGF2BP2* gene with the risk of BCR and clinicopathological progression in Taiwanese PCa patients who had undergone an RP.

## Materials and Methods

### Study participants

This study involved the analysis of blood samples from 698 PCa patients who underwent a robotic-assisted laparoscopic RP at Taichung Veterans General Hospital (TVGH; Taichung, Taiwan), between 2012 and 2018. Written informed consent was obtained from all participants before venous blood collection, and the study protocol received approval from the TVGH Institutional Review Board (IRB no. CE19062A-2). Medical data collected at the time of diagnosis included PSA levels, pathologic Gleason grades, clinical and pathologic T (tumor) and N (node) staging, cancer invasion areas (seminal vesicle, perineural, and lymphovascular regions), D'Amico classification, and the BCR status.

### Genomic DNA extraction

Whole-blood samples from PCa patients were collected in EDTA-containing tubes. Genomic DNA was then extracted from the buffy coat layer following centrifugation, using a QIAamp DNA Blood Mini Kit (Qiagen, Valencia, CA, USA) according to the previously described^27^. The quality of the extracted DNA was assessed with a Nanodrop-2000 spectrophotometer (Thermo Scientific, Waltham, MA, USA) before serving as a template for a polymerase chain reaction (PCR)^28^.

### Selection of *IGF2BP2* SNPs

Three SNPs located in the second intron of *IGF2BP2*—rs11705701 (G/A), rs4402960 (G/T), and rs1470579 (A/C)—were selected for analysis in this study. These SNPs were chosen based on prior studies highlighting rs4402960 and rs1470579 as the most prevalent variants associated with diabetes and various cancers^17^. Additionally, rs11705701 was linked to an increased risk of T2D^29^ and oral cancer progression^25^.

### Determination of *IGF2BP2* SNPs

The TaqMan SNP Genotyping Assay on an ABI StepOnePlus™ Real-Time PCR System (Applied Biosystems, Foster City, CA, USA) was used to identify alleles of *IGF2BP2* SNPs—rs11705701 (assay ID: C_31742122_10), rs4402960 (assay ID: C_2165199_10), and rs1470579 (assay ID: C_2165184_10). Results were analyzed using SDS vers. 3.0 software (Applied Biosystems, Foster City, CA, USA). Detailed procedures of DNA genotyping were outlined in our previous study^30^.

### Bioinformatics analysis

Clinical data and mRNA sequencing for prostate adenocarcinoma (PRAD) samples from The Cancer Genome Atlas (TCGA) were accessed via the UCSC Xena database. *IGF2BP2* gene expression was analyzed in relation to clinical features, including Gleason scores and clinical M stages. Progression-free survival (PFS) was assessed by categorizing PRAD patients into high- and low-IGF2BP2 expression groups, with statistical significance determined using the log-rank test. To identify IGF2BP2-associated pathways, a gene set enrichment analysis (GSEA) was performed.

### Statistical analysis

Chi-squared and Student's *t*-test were employed to compare demographic characteristics between the PSA ≤ 10 ng/mL and > 10 ng/mL groups. Odds ratios (ORs) and adjusted ORs (AORs) with 95% confidence intervals (CIs) were estimated using multiple logistic regression models to determine associations between genotypic frequencies and the two PSA groups, as well as the risk of different clinicopathological characteristics. All statistical analyses were performed using SAS software (vers. 9.1, 2005, for Windows; SAS Institute, Cary, NC, USA), with* p* values of < 0.05 considered statistically significant.

## Results

### Demographic characteristics of PCa patients with high and low iPSA levels

Table 1 compares demographic characteristics of PCa patients with high iPSA (> 10 ng/mL, 365 patients) and low iPSA (≤ 10 ng/mL, 333 patients). Patients with a high iPSA level were significantly more likely to present with advanced clinical T stages (T3+T4) and N stage (N1) at diagnosis compared to those with a low iPSA. Surgical pathological findings showed that high-iPSA patients more frequently exhibited higher pathologic Gleason grades (4+5), advanced pathologic T (T3+T4) and N (N1) stages, and greater evidence of seminal vesicle, perineural, and lymphovascular invasion. Additionally, based on the D'Amico risk classification, a larger proportion of high-iPSA patients fell into the high-risk category. Furthermore, these patients also demonstrated a higher recurrence rate.

### Associations between *IGF2BP2* SNPs and iPSA levels in PCa patients

We next investigated the potential impacts of the three selected *IGF2BP2* SNPs—rs11705701 (G/A), rs4402960 (G/T), and rs1470579 (A/C)—on iPSA levels in PCa patients at diagnosis. Genotype frequencies of these SNPs were analyzed in a cohort of 698 PCa patients. The most prevalent alleles were homozygous G/G for rs11705701 and rs4402960, and homozygous A/A for rs1470579 (Table 2). After adjusting for potential confounders, no significant associations were identified of the polymorphic frequencies of rs11705701, rs4402960, and rs1470579 with iPSA levels.

### Relationships between clinicopathological features and *IGF2BP2* SNPs in PCa patients with high and low iPSA levels

Next, to clarify impacts of *IGF2BP2* genetic polymorphisms on PCa clinicopathological characteristics, we analyzed factors such as pathologic staging, clinical staging, Gleason grade groups, tumor invasion, D'Amico classification, and BCR. Among the three IGF2BP2 loci studied, the rs1470579 AC genotype was associated with a significantly higher risk of PNI (OR = 1.455, 95% CI = 1.009-2.100; *p* = 0.044) compared to the wild-type (WT) AA genotype, as shown in Table 3. In contrast, no significant associations were identified for rs11705701 or rs4402960 with the clinicopathological features analyzed (data not shown). Further stratification of PCa patients into high (*n* = 365) and low (*n* = 333) iPSA groups revealed that the rs1470579 AC or AC+CC genotypes had significantly higher risks of PNI in patients with high iPSA levels (OR = 1.862, 95% CI = 1.049-3.302; *p* = 0.032 and OR = 1.756, 95% CI = 1.022-3.017, respectively) (Tables 4, 5). However, this association was not observed in patients with low iPSA levels (data not shown).

### Associations of IGF2BP2 expression levels with clinicopathological characteristics and prognoses of PCa patients

**Figure 1:**
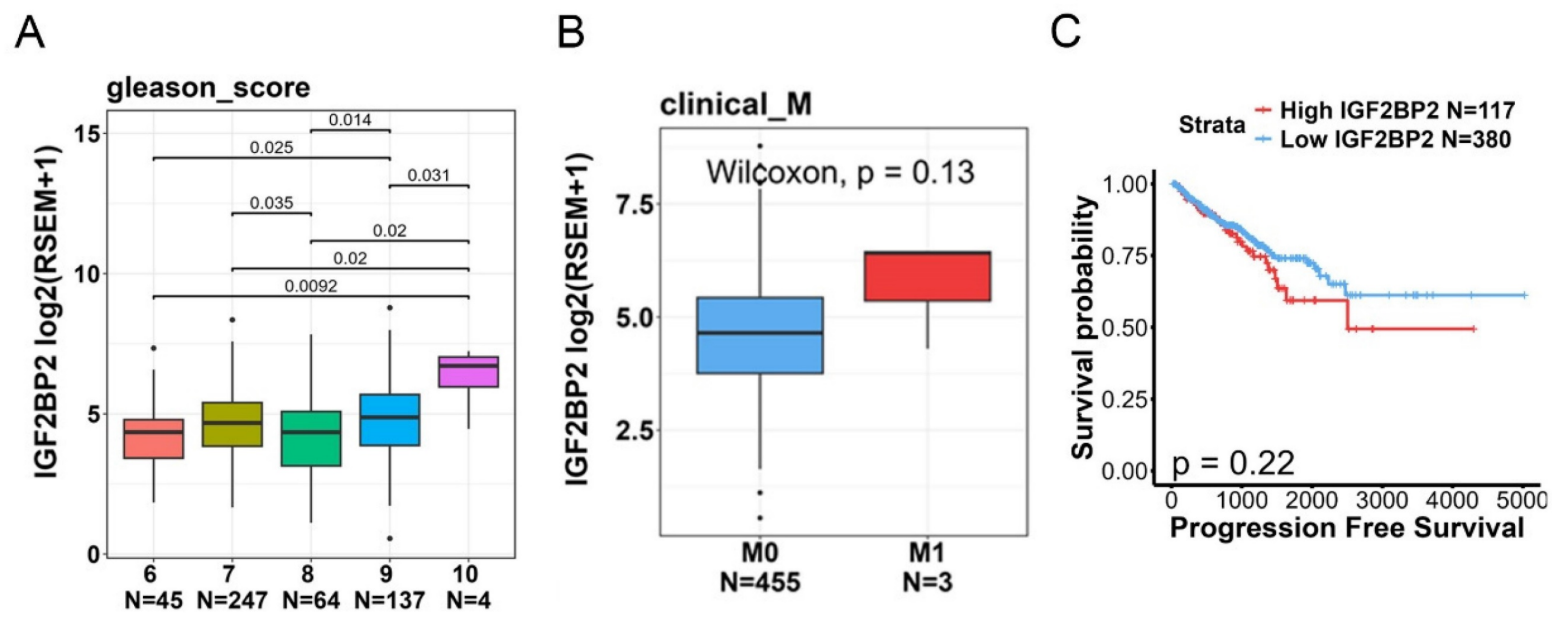
Clinical significance of IGF2BP2 expression in prostate cancer (PCa) patients evaluated using data from TCGA-prostate adenocarcinoma (PRAD) dataset. (A, B) IGF2BP2 expression levels were analyzed and compared according to Gleason scores (A) and clinical M stages (B) within TCGA-PRAD dataset. (C) Kaplan-Meier survival curves illustrating progression-free survival in patients with high versus low IGF2BP2 expression levels.

To further explore correlations between IGF2BP2 levels and disease progression or prognosis, we utilized TCGA-PRAD dataset. Our analysis revealed that IGF2BP2 expression was significantly higher in patients with high Gleason scores (Figure 1A). Moreover, we observed a trend where tumor tissues with distal metastasis exhibited higher IGF2BP2 expression compared to those without metastasis (Figure 1B). A Kaplan-Meier plot indicated that a higher IGF2BP2 expression level showed a trend to be associated with a shorter duration of PFS (Figure 1C).

### Exploration of the potential molecular mechanisms mediated by IGF2BP2 in PCa progression

**Figure 2:**
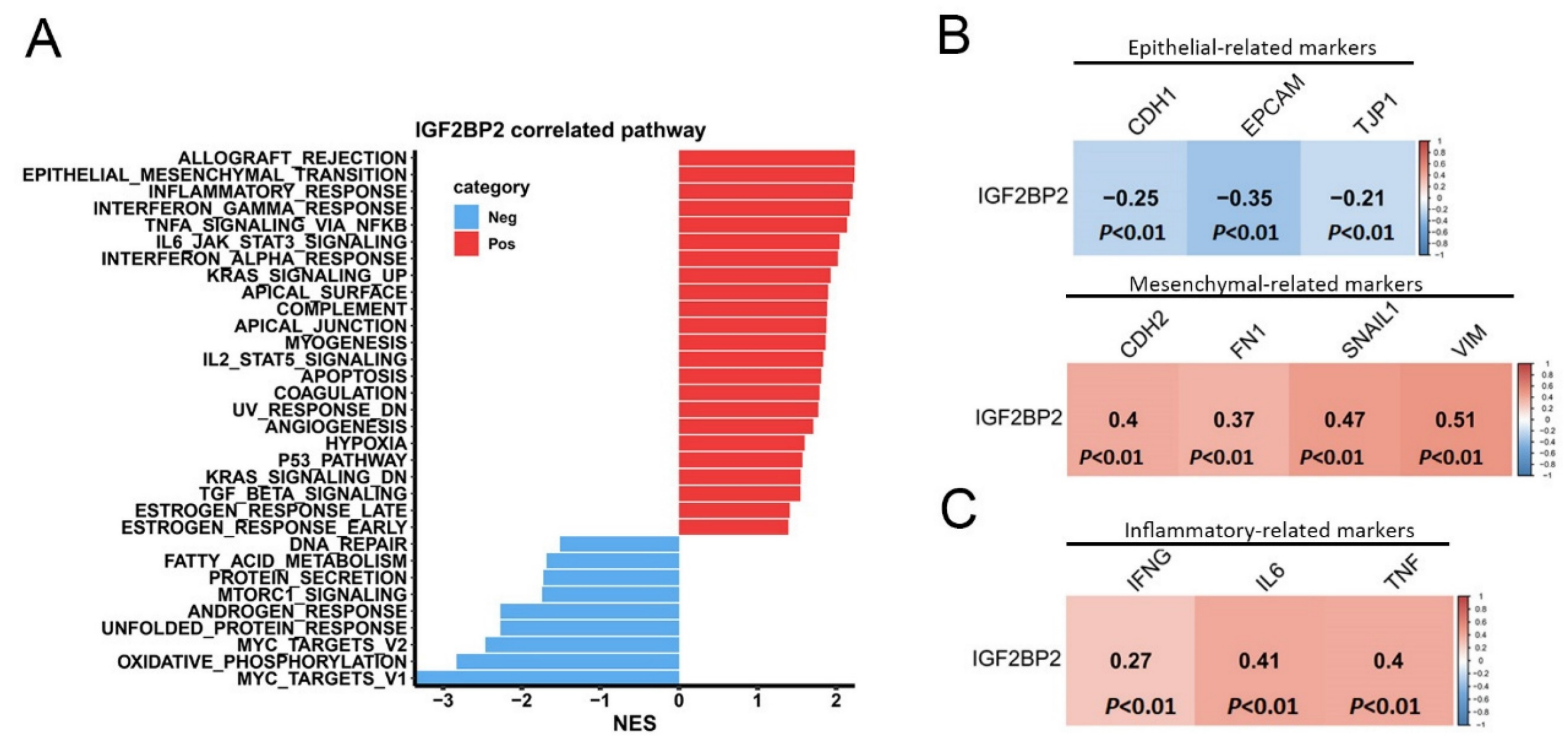
Pathways associated with IGF2BP2 in prostate cancer (PCa) patients. (A) Horizontal bar plot illustrating pathways linked to IGF2BP2 expression. Pathways positively associated with IGF2BP2 are shown in red, while those negatively associated are in blue. The x-axis displays normalized enrichment scores (NESs), and the y-axis lists pathways identified from the Hallmark database. (B, C) Correlation plots showing relationships of IGF2BP2 expression with biomarkers of the epithelial-mesenchymal transition (B) and with biomarkers of inflammatory responses (C). RNA sequencing data from TCGA prostate adenocarcinoma (PRAD) dataset were analyzed. A Pearson correlation analysis was conducted to assess relationships between IGF2BP2 and the biomarkers, with correlation coefficients and *p* values displayed in each square. The scale bar indicates the strength of the correlation.

In order to investigate the mechanisms through which IGF2BP2 contributes to PCa progression, we performed a gene set enrichment analysis (GSEA) using TCGA-PRAD dataset. Results revealed that the epithelial-mesenchymal transition (EMT) pathway exhibited the second highest NES in association with IGF2BP2 expression (Figure 2A). Notably, the EMT was closely linked to PNI, as elevated levels of EMT regulators such as transforming growth factor-β (TGFB) and serpin peptidase inhibitor, clade E (nexin, plasminogen activator inhibitor type 1), member 1 (SERPINE1) are frequently detected in PNI-associated tissues^31^. Furthermore, the analysis identified several inflammation-related Hallmark gene sets as being enriched in patients with high IGF2BP2 expression (Figure 2A). Chronic inflammation was implicated as a key factor in PCa development and its progression to advanced metastatic stages^32^. Using the cBioPortal platform, we further analyzed correlations between EMT-related markers and IGF2BP2 expression in PCa tissues from TCGA. A strong positive correlation was observed between *IGF2BP2* and mesenchymal phenotype-related genes (*CDH2, FN1, SNAI1,* and *VIM*) (Figure 2B, lower panel). Conversely, epithelial phenotype-related genes (*CDH1, TJP1,* and *EPCAM*) were negatively correlated with *IGF2BP2* expression (Figure 2B, upper panel). Additionally, IGF2BP2 expression was positively associated with inflammation-related genes (*IFNG, IL6,* and *TNF*) (Figure 2C).

## Discussion

Given the pivotal role of IGF2BP2 as an m6A reader that stabilizes RNA and promotes oncogenic effects in PCa progression^33^, ^34^, we examined polymorphisms in the second intron of the *IGF2BP2* gene, observing distinct distributions in PCa patients with high versus low iPSA levels. Our analysis revealed that patients carrying the mutant AC genotype of rs1470579 exhibited a significantly elevated risk of developing PNI, with stronger associations observed in those with high iPSA. These findings underscore the potential impact of specific *IGF2BP2* genetic variants on PNI, particularly in high-iPSA PCa patients at diagnosis. Additionally, we found that IGF2BP2 expression levels were significantly associated with pathological Gleason scores, and EMT- and inflammatory-related pathways in PCa patients.

Previous studies showed that PCa patients with PNI tend to have higher iPSA levels compared to those without PNI. Additionally, PNI was linked to elevated surgical Gleason scores^5^. We suggest that the rs1470579 SNP may regulate IGF2BP2 expression, thereby promoting PNI in PCa patients, particularly those with high iPSA. Consistent with this, we observed that IGF2BP2 expression was also associated with higher Gleason scores in PCa tissues. The rs1470579 SNP is situated within the intron of the *IGF2BP2* gene. Although polymorphisms in intronic regions do not directly alter protein sequences, growing evidence indicates that such variations can lead to splicing abnormalities, potentially affecting translation and contributing to various diseases, including cancers^35^. Moreover, intronic sequences often contain numerous cis-acting regulatory elements (CREs), such as transcription factor-binding sites, enhancers, and silencers, which can modulate gene expression either positively or negatively^36^. Additionally, many lncRNAs are embedded within intronic regions and are known to regulate expressions of their corresponding host genes^36^. In this study, we proposed that the *IGF2BP2* SNP rs1470579 may affect IGF2BP2 expression, contributing to the development of PNI in PCa. However, the mechanism by which the rs1470579 SNP regulates IGF2BP2 expression remains uncertain and requires further exploration in future research.

Recent studies suggested a possible role of the EMT in PNI of tumor cells, including salivary gland cystic carcinoma (SACC). The brain-derived neurotrophic factor (BDNF)/tropomyosin-related kinase B (TrkB) axis was shown to facilitate tumor progression and PNI through the EMT in SACC^37^. Similarly, in PCa, activation of the BDNF/TrkB pathway was implicated in disease progression by inducing the EMT^38^. EMT regulators are emerging as potential biomarkers for PNI. For instance, silencing the EMT-inducing factor, Slug, was demonstrated to increase E-cadherin expression, thereby inhibiting the EMT and PNI in SACC^39^. Additionally, Kakies *et al.* reported a PCa case featuring neuroendocrine differentiation and extensive PNI, in which reduced E-cadherin and elevated vimentin levels highlighted potential links of neuroendocrine differentiation with the, EMT and PNI in PCa^7^. In this study, we identified a positive association between *IGF2BP2* expression and EMT-related gene signatures in TCGA-PRAD dataset. Analysis of human PCa samples using the cBioPortal platform revealed that *IGF2BP2* expression was significantly correlated with mesenchymal phenotype-related genes and inversely correlated with epithelial phenotype-related genes. Furthermore, IGF2BP2 expression was shown to promote the EMT and metastasis in various cancers, including gastric^40^ and oral^41^ cancers. These findings suggest that the rs1470579 genetic variant might upregulate IGF2BP2 expression, influence the EMT process, and thereby contribute to PNI in PCa.

Several tumor studies reported a strong association between nerve invasion and an inflammatory response^42^, ^43^. Additionally, nerve invasion was implicated in facilitating immune escape during tumor development^44^. These observations highlight a close interplay among PNI, inflammation, and immune responses. In this study, we found that IGF2BP2 expression was significantly associated with multiple inflammation-related pathways in PCa, including INFLAMMATORY_RESPONSE, INTERFERON_GAMMA_RESPONSE, TNFA_SIGNALING_VIA_NFKB, and IL6_JAK_STAT3_SIGNALING. Analysis of TCGA dataset further revealed notable correlations between IGF2BP2 expression and levels of key inflammatory mediators, such as interferon-γ (IFNG), interleukin-6 (IL6), and tumor necrosis factor (TNF), in PCa tissues. Moreover, previous research demonstrated that IGF2BP2-knockdown suppressed inflammatory responses in gastric cancer^45^. These findings suggest that the rs1470579 genetic variant may enhance IGF2BP2 expression, thereby modulating interactions between inflammatory responses and PNI in PCa.

In summary, this is the first study to investigate distinct allelic effects of the IGF2BP2 rs1470579 SNP in a Taiwanese population, emphasizing its influence on the occurrence of PNI in PCa. Our findings suggest that IGF2BP2-related pathways, including the EMT and inflammatory responses, may act as key contributors to PCa-associated PNI. Additionally, the IGF2BP2 rs1470579 variant holds promise as a potential biomarker for PNI in PCa, particularly in patients presenting with high iPSA levels.

## Figures and Tables

**Table 1:** Distributions of demographic characteristics among 698 prostate cancer patients

Variable	PSA at diagnosis (ng/mL)			
	≤ 10 (*N*=333)	> 10 (*N*=365)		*p* value
Age at diagnosis (years)				
≤ 65	159 (47.7 %)		136 (37.3 %)	*p*=0.005*
> 65	174 (52.3 %)		229 (62.7 %)	
Pathologic Gleason grade group				
1+2+3	303 (91.0 %)		277 (75.9 %)	*p*<0.001*
4+5	30 (9.0 %)		88 (24.1 %)	
Clinical T stage				
1+2	313 (94.0 %)		290 (78.8 %)	*p*<0.001*
3+4	20 (6.0 %)		75 (20.5 %)	
Clinical N stage				
N0	330 (99.1 %)		354 (97.0 %)	*p*=0.047*
N1	3 (0.9 %)		11 (3.0 %)	
Pathologic T stage				
2	230 (69.1 %)		141 (38.6 %)	*p*<0.001*
3+4	103 (30.9 %)		224 (61.4 %)	
Pathologic N stage				
N0	318 (95.5 %)		320 (87.7 %)	*p*<0.001*
N1	15 (4.5 %)		45 (12.3 %)	
Seminal vesicle invasion				
No	302 (90.7 %)		248 (67.9 %)	*p*<0.001*
Yes	31 (9.3 %)		117 (32.1 %)	
Perineural invasion				
No	114 (34.2%)		72 (19.7 %)	*p*<0.001*
Yes	219 (65.8 %)		293 (80.3 %)	
Lymphovascular invasion				
No	306 (91.9 %)		281 (77.0 %)	*p*<0.001*
Yes	27 (8.1 %)		84 (23.0 %)	
D'Amico classification				
Low/Intermediate risk	239 (71.8 %)		107 (29.3 %)	*p*<0.001*
High risk	94 (28.2 %)		258 (70.7 %)	
Biochemical recurrence				
No	266 (79.9 %)		211 (57.8 %)	*p*<0.001*
Yes	67 (20.1 %)		154 (42.2 %)	
				

* *p* < 0.05 indicates statistical significance. PSA, prostate-specific antigen.

**Table 2:** Distribution frequencies of *IGF2BP2* genotypes in 698 prostate cancer patients with high or low initial prostate-specific antigen (PSA)

Variable	PSA at diagnosis (ng/mL)			
	≤ 10 (*N*=333) (%)	> 10 (*N*=365) (%)	AOR (95% CI)	*p* value
rs11705701				
GG	194 (58.3%)	225 (61.6%)	1.000	
GA	118 (35.4%)	126 (34.5%)	0.914 (0.637~1.313)	*p*=0.628
AA	21 (6.3%)	14 (3.8%)	0.625 (0.284~1.375)	*p*=0.242
GA+AA	139 (41.7%)	140 (38.3%)	0.871 (0.616~1.232)	*p*=0.435
rs4402960				
GG	188 (56.5%)	216 (59.2%)	1.000	
GT	119 (35.7%)	134 (36.7%)	0.902 (0.630~1.293)	*p*=0.575
TT	26 (7.8%)	15 (4.1%)	0.591 (0.281~1.245)	*p*=0.167
GT+TT	145 (43.5%)	149 (40.8%)	0.850 (0.603~1.198)	*p*=0.354
rs1470579				
AA	184 (55.3%)	204 (55.9%)	1.000	
AC	123 (36.9%)	141 (38.6%)	0.949 (0.663~1.357)	*p*=0.773
CC	26 (7.8%)	20 (5.5%)	0.710 (0.351~1.437)	*p*=0.341
AC+CC	149 (44.7%)	161 (44.1%)	0.909 (0.646~1.279)	*p*=0.583

The odds ratios (ORs) and their 95% confidence intervals (CIs) were estimated by logistic regression models. The adjusted ORs (AORs) with their 95% CIs were estimated by multiple logistic regression models after controlling for age at diagnosis, pathologic Gleason grade group, clinical T stage, pathologic T stage, pathologic N stage, seminal vesicle invasion, perineural invasion, lymphovascular invasion, biochemical recurrence, and D'Amico classification.

**Table 3:** Odds ratios (ORs) and 95% confidence intervals (CIs) for associations between clinical characteristics and genotypic distributions of *IGF2BP2* rs1470579 in a cohort of 698 prostate cancer patients

Variable	rs1470579						
	AA (*N*=388)	AC (*N*=264)	CC (*N*=46)	AA vs AC OR (95% CI)	*p* value	AA vs CC OR (95% CI)	*p* value
Pathologic Gleason grade group							
1+2+3	321 (82.7%)	220 (83.3%)	39 (84.8%)	1.000	0.841	1.000	0.727
4+5	67 (17.3%)	44 (16.7%)	7 (15.2%)	0.958 (0.631~1.454)		0.860 (0.369~2.005)	
Clinical T stage							
1+2	329 (84.8%)	232 (87.9%)	42 (91.3%)	1.000	0.265	1.000	0.236
3+4	59 (15.2%)	32 (12.1%)	4 (8.7%)	0.769 (0.485~1.221)		0.531 (0.184~1.537)	
Clinical N stage							
N0	377 (97.2%)	262 (99.2%)	45 (97.8%)	1.000	0.062	1.000	0.796
N1	11 (2.8%)	2 (0.8%)	1 (2.2%)	0.262 (0.058~1.190)		0.762 (0.096~6.038)	
Pathologic T stage							
2	214 (55.2%)	128 (48.5%)	29 (63.0%)	1.000	0.094	1.000	0.308
3+4	174 (44.8%)	136 (51.5%)	17 (37.0%)	1.307 (0.955~1.788)		0.721 (0.384~1.355)	
Pathologic N stage							
N0	353 (91.0%)	241 (91.3%)	44 (95.7%)	1.000	0.892	1.000	0.283
N1	35 (9.0%)	23 (8.7%)	2 (4.3%)	0.963 (0.555~1.670)		0.458 (0.107~1.972)	
Seminal vesicle invasion							
No	310 (79.9%)	200 (75.8%)	40 (87.0%)	1.000	0.209	1.000	0.252
Yes	78 (20.1%)	64 (24.2%)	6 (13.0%)	1.272 (0.874~1.851)		0.596 (0.244~1.456)	
Perineural invasion							
No	111 (28.6%)	57 (21.6%)	18 (39.1%)	1.000	0.044*	1.000	0.140
Yes	277 (71.4%)	207 (78.4%)	28 (60.9%)	1.455 (1.009~2.100)		0.623 (0.331~1.172)	
Lymphovascular invasion							
No	326 (84.0%)	221 (83.7%)	40 (87.0%)	1.000	0.916	1.000	0.604
Yes	62 (16.0%)	43 (16.3%)	6 (13.0%)	1.023 (0.669~1.565)		0.789 (0.321~1.940)	
D'Amico classification							
Low risk/Intermediate risk	194 (50.0%)	128 (48.5%)	24 (52.2%)	1.000	0.704	1.000	0.780
High risk	194 (50.0%)	136 (51.5%)	22 (47.8%)	1.063 (0.777~1.453)		0.917 (0.497~1.690)	
Biochemical recurrence							
No	268 (69.1%)	177 (67.0%)	32 (69.6%)	1.000	0.585	1.000	0.945
Yes	120 (30.9%)	87 (33.0%)	14 (30.4%)	1.098 (0.785~1.535)		0.977 (0.503~1.898)	

ORs with their 95% CIs were estimated by logistic regression models. * *p* < 0.05 indicates statistical significance.

**Table 4:** Odds ratios (ORs) and 95% confidence intervals (CIs) of relationships between clinical characteristics and *IGF2BP2* rs1470579 single-nucleotide polymorphisms under a codominant model in 365 prostate cancer patients with prostate-specific antigen of >10 ng/mL

Variable	rs1470579						
	AA (*N*=204)	AC (*N*=141)	CC (*N*=20)	AA vs AC OR (95% CI)	*p* value	AA vs CC OR (95% CI)	*p* value
Pathologic Gleason grade group							
1+2+3	154 (75.5%)	107 (75.9%)	16 (80.0%)	1.000	0.993	1.000	0.653
4+5	50 (24.5%)	34 (24.1%)	4 (20.0%)	0.979 (0.593~1.615)		0.770 (0.246~2.410)	
Clinical T stage							
1+2	162 (79.4%)	111 (78.7%)	17 (85.0%)	1.000	0.877	1.000	0.552
3+4	42 (20.6%)	30 (21.3%)	3 (15.0%)	1.042 (0.615~1.766)		0.681 (0.190~2.432)	
Clinical N stage							
N0	195 (95.6%)	140 (99.3%)	19 (95.0%)	1.000	0.091	1.000	0.903
N1	9 (4.4%)	1 (0.7%)	1 (5.0%)	0.155 (0.019~1.236)		1.140 (0.137~9.491)	
Pathologic T stage							
2	86 (42.2%)	48 (34.0%)	7 (35.0%)	1.000	0.128	1.000	0.535
3+4	118 (57.8%)	93 (66.0%)	13 (65.0%)	1.412 (0.904~2.205)		1.354 (0.518~3.535)	
Pathologic N stage							
N0	179 (87.7%)	123 (87.2%)	18 (90.0%)	1.000	0.888	1.000	0.768
N1	25 (12.3%)	18 (12.8%)	2 (10.0%)	1.048 (0.548~2.003)		0.796 (0.174~3.636)	
Seminal vesicle invasion							
No	144 (70.6%)	89 (63.1%)	15 (75.0%)	1.000	0.145	1.000	0.678
Yes	60 (29.4%)	52 (36.9%)	5 (25.0%)	1.402 (0.889~2.212)		0.800 (0.278~2.300)	
Perineural invasion							
No	48 (23.5%)	20 (14.2%)	4 (20.0%)	1.000	0.032*	1.000	0.721
Yes	156 (76.5%)	121 (85.8%)	16 (80.0%)	1.862 (1.049~3.302)		1.231 (0.393~3.858)	
Lymphovascular invasion							
No	159 (77.9%)	107 (75.9%)	15 (75.0%)	1.000	0.655	1.000	0.763
Yes	45 (22.1%)	34 (24.1%)	5 (25.0%)	1.123 (0.675~1.867)		1.178 (0.406~3.416)	
D'Amico classification							
Low risk/Intermediate risk	66 (32.4%)	36 (25.5%)	5 (25.0%)	1.000	0.172	1.000	0.500
High risk	138 (67.6%)	105 (74.5%)	15 (75.0%)	1.395 (0.864~2.252)		1.435 (0.500~4.116)	
Biochemical recurrence							
No	123 (60.3%)	77 (54.6%)	11 (55.0%)	1.000	0.293	1.000	0.645
Yes	81 (39.7%)	64 (45.4%)	9 (45.0%)	1.262 (0.818~1.948)		1.242 (0.493~3.132)	

ORs with their 95% CIs were estimated by logistic regression models. * *p* < 0.05 indicates statistical significance.

**Table 5:** Odds ratios (ORs) and 95% confidence intervals (CIs) for the relationship between clinical characteristics and *IGF2BP2* rs1470579 single-nucleotide polymorphisms under a dominant model in 365 prostate cancer patients with prostate-specific antigen of >10 ng/mL

Variable	Genotypic frequencies			
rs1470579	AA (*N*=204)	AC+CC (*N*=161)	OR (95% CI)	*p* value
Pathologic Gleason grade group				
1+2+3	154 (75.5%)	123 (76.4%)	1.000	0.841
4+5	50 (24.5%)	38 (23.6%)	0.952 (0.587~1.544)	
Clinical T stage				
1+2	162 (79.4%)	128 (79.5%)	1.000	0.983
3+4	42 (20.6%)	33 (20.5%)	0.994 (0.596~1.658)	
Clinical N stage				
N0	195 (95.6%)	159 (98.8%)	1.000	0.079
N1	9 (4.4%)	2 (1.2%)	0.273 (0.058~1.279)	
Pathologic T stage				
2	86 (42.2%)	55 (34.2%)	1.000	0.119
3+4	118 (57.8%)	106 (65.8%)	1.405 (0.915~2.155)	
Pathologic N stage				
N0	179 (87.7%)	141 (87.6%)	1.000	0.961
N1	25 (12.3%)	20 (12.4%)	1.016 (0.542~1.903)	
Seminal vesicle invasion				
No	144 (70.6%)	104 (64.6%)	1.000	0.223
Yes	60 (29.4%)	57 (35.4%)	1.315 (0.846~2.046)	
Perineural invasion				
No	48 (23.5%)	24 (14.9%)	1.000	0.040*
Yes	156 (76.5%)	137 (85.1%)	1.756 (1.022~3.017)	
Lymphovascular invasion				
No	159 (77.9%)	122 (75.8%)	1.000	0.626
Yes	45 (22.1%)	39 (24.2%)	1.130 (0.692~1.843)	
D'Amico classification				
Low risk/Intermediate risk	66 (32.4%)	41 (25.5%)	1.000	0.151
High risk	138 (67.6%)	120 (74.5%)	1.400 (0.884~2.218)	
Biochemical recurrence				
No	123 (60.3%)	88 (54.7%)	1.000	0.279
Yes	81 (39.7%)	73 (45.3%)	1.260 (0.829~1.914)	

ORs with their 95% CIs were estimated by logistic regression models. * *p* < 0.05 indicates statistical significance.
